# Supplementary material for: Clonal behaviour of myogenic precursor cells throughout the vertebrate lifespan
Source: Biol Open. 2022 Aug 16;11(8):bio059476. doi: 10.1242/bio.059476 (PMC9399818; doi:10.1242/bio.059476)
Supplement: Supplementary information [file biolopen-11-059476-s1.pdf]

Fig. S1

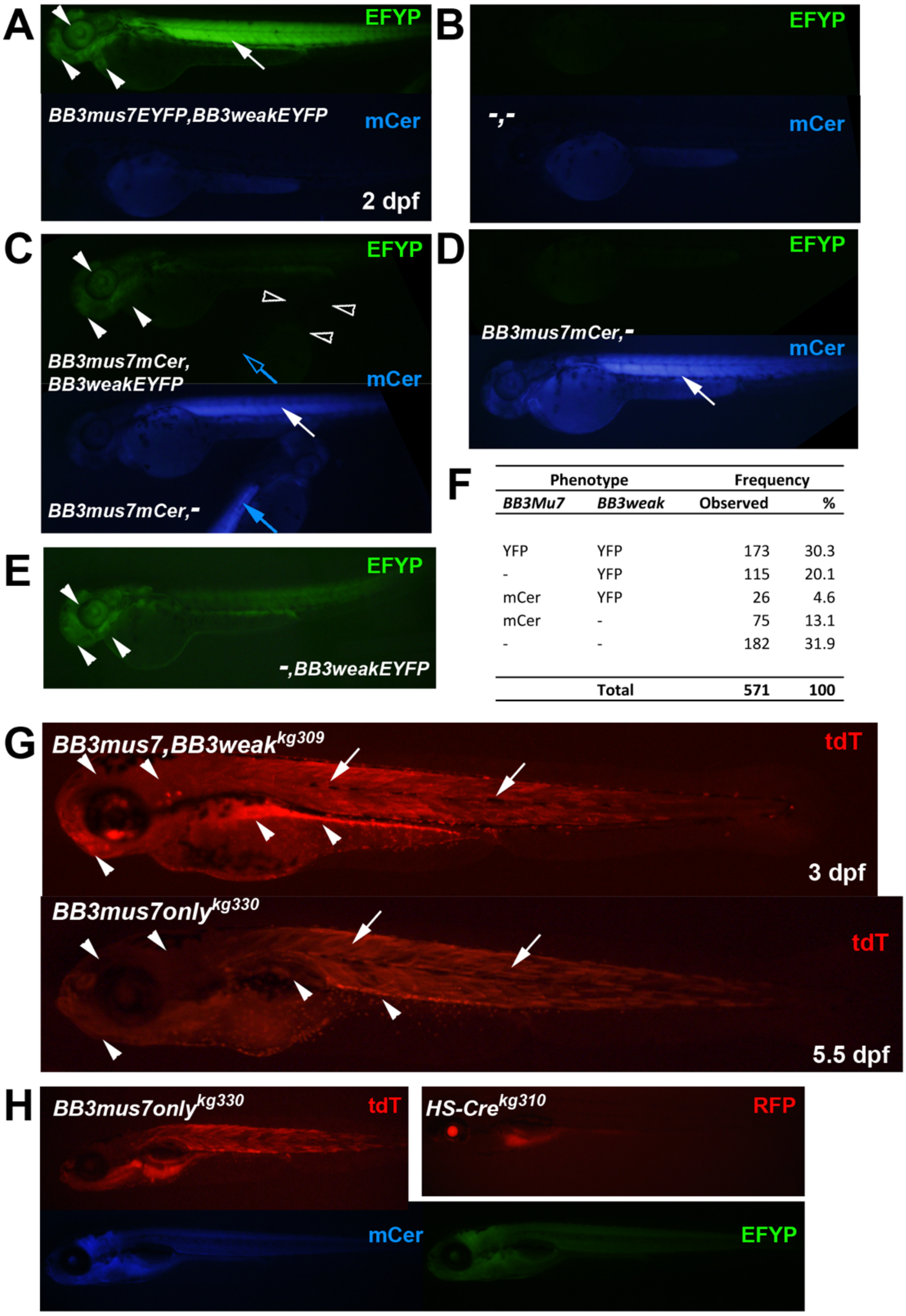

Phenotype		Frequency	
BB3Mu7	*BB3weak*	Observed	%
YFP	YFP	173	30.3
-	YFP	115	20.1
mCer	YFP	26	4.6
mCer	-	75	13.1
-	-	182	31.9
	Total	571	100

Fig. S1. Generation of the *kg330* allele. Linked BB3 insertions in *kg309* yield distinct patterns of recombination. When four independent female *BB3mus7*$^{kg309/+}$; *HS:Cre.cryaa:RFP*^{kg310} fish were outcrossed to wild type fish, only 182/571

Biology Open • Supplementary information

(32%) of progeny lacked fluorescence (excluding lens RFP), a highly significant difference from both the predicted 50% for a single transgene insertion (p = 9.0E-10, C2 test) and the 25% expected for two (or <25% for more than two) separate unlinked transgenes (p = 1.5E-10 4, C^2 test) indicating two linked BB3 insertions in the *BB3mus7*^{kg309} allele. Female germline Cre expression leads to non-mosaic recombination yielding several distinct patterns of fluorescent protein expression at each linked BB3 locus. **A**. Recombination of the *BB3mus7* transgene yielded strong myotomal EYFP expression (arrow). A second separable insertion yielded widespread low-level EYFP (*BB3weak*) expression in most tissues including brain, eye, heart, epidermis and gut (arrowheads). Such fish never express mCer (lower panel). **B.** Many fish lacked fluorescence altogether, revealing the background autofluorescence level. **C,D.** Less frequently, individuals recombined at *BB3mus7* to give myotomal mCer expression (arrows) either alone (C lower fish and D) or in combination with *BB3weak* EYFP (C upper fish). Note the presence of *BB3weak* signal (filled arrowheads) in head of the upper individual, but not of the lower individual (open arrowheads) and *BB3mus7* mCer in both upper (arrow) and lower (blue arrows) larvae. **E.** Individuals with only the *BB3weak* EFYP were numerous. Note the contrast of the weak signal in trunk in this enhanced imaged compared with the *BB3mus7EYFP,BB3weakEYFP* transgenic in panel A. **F.** Frequency of each genotype indicating genetic linkage and recombination dynamics. Note that *BB3mus7* transmitted to 274/571 (48%) of progeny, as expected for a single heterozygous Mendelian locus (p = 0.34, 26 X^2 test). Lack of mCer recombination at the *BB3weak* locus suggests genetic interference, supporting linkage of *BBSmus7* and *BB3weak.* **G.** Outcrossing of *kg309* (tdT expression shown in the upper individual) permitted separation on the basis of the tdTomato of *BB3mus7* and *BB3weak* to isolate the *BB3mus7only kg330* allele (lower larva). Note the widespread weak tdT signal in head and gut of *BB3mus7*^{kg309} (arrowheads) and its reduction in *BB3mus7*^{kg330} compared to the *BB3mus7* myotomal signal (arrows). **H.** Lateral views of single *BB3mus7*^{kg330} and *HS:Cre.cryaa:RFP*^{kg310} fish at 6 dpf showing the distinct RFP and tdT signal and the low mCer and EYFP background in somites of unrecombined Musclebow2.

Biology Open • Supplementary information

Fig S2

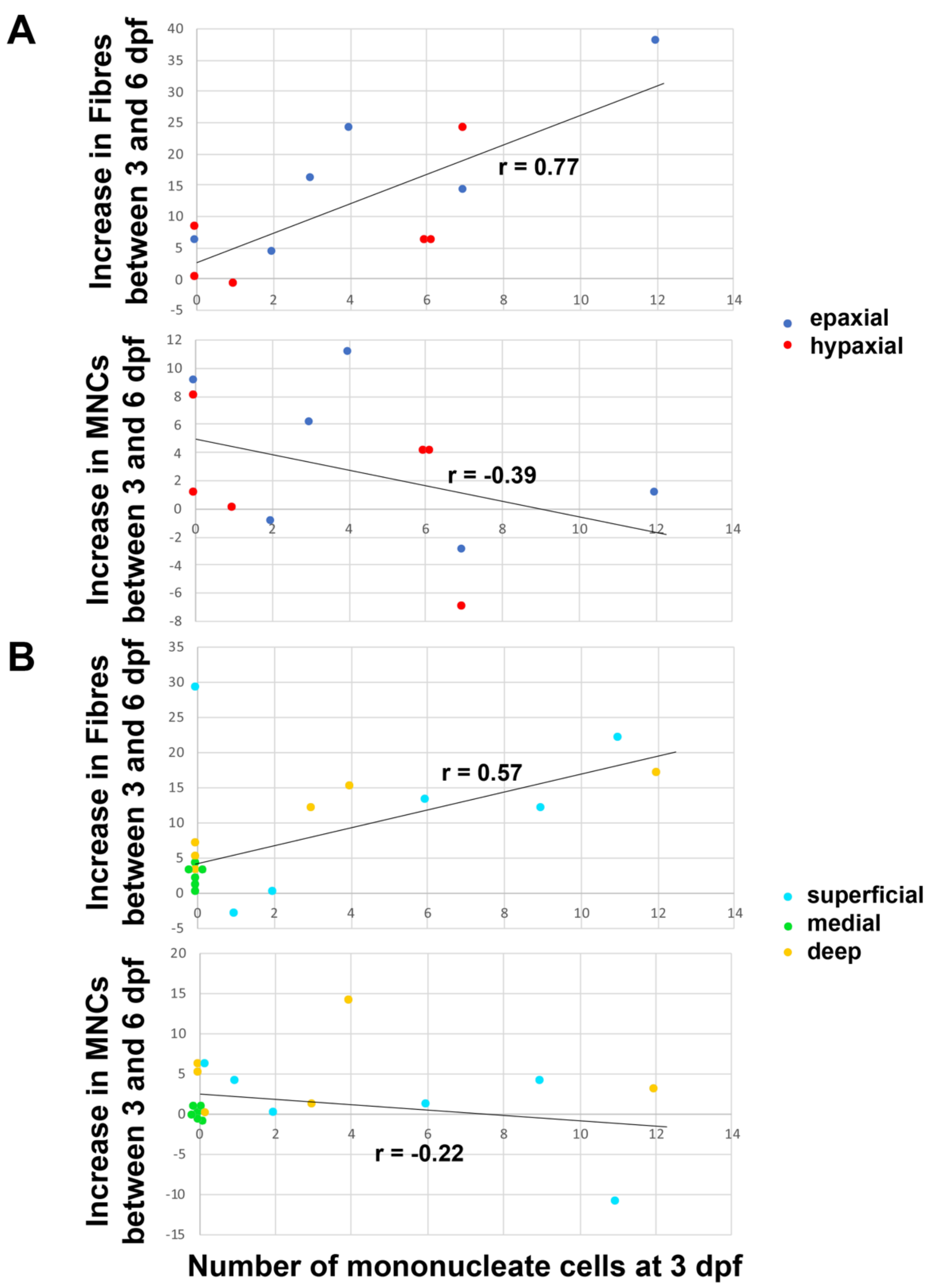

Fig. S2. Regional correlation of myogenesis from marked mncs. Change in number of marked fibres (upper graphs) and mncs (lower charts) in each region of six single somites analysed at both 3 and 6 dpf, from same dataset shown in Fig. 3C. **A.** Similar behaviour in epaxial and hypaxial somitic regions. **B.** Dispersion of largely superficial mncs into deeper regions. Colours indicate final location at 6 dpf.

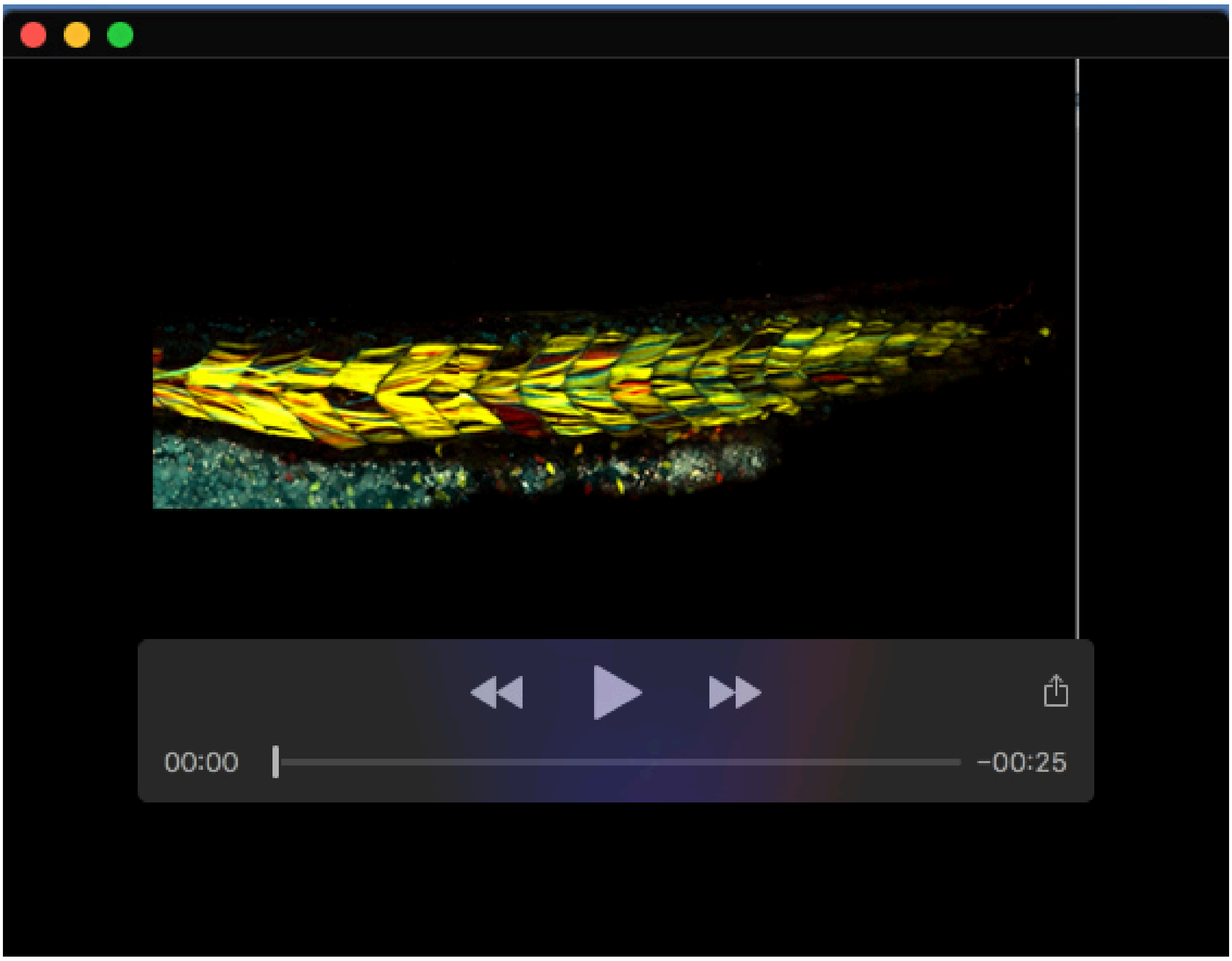

Movie 1. Stability of Musclebow2 labelling on short timescales.
Tg(BB3mus7)^{kg309}*;Tg(HS:Cre.cryaa:RFP)*^{kg310} embryo was subjected to 5 min heat-shock at 30% epiboly. After embedding in 3% methylcellulose with light tricaine at 26 hpf, 15 sequential confocal time-lapse 3-colour 3-tile 2 μm stacks were taken every 24 min over 6 h at 24°C. An 11-slice mip of each stack is shown.

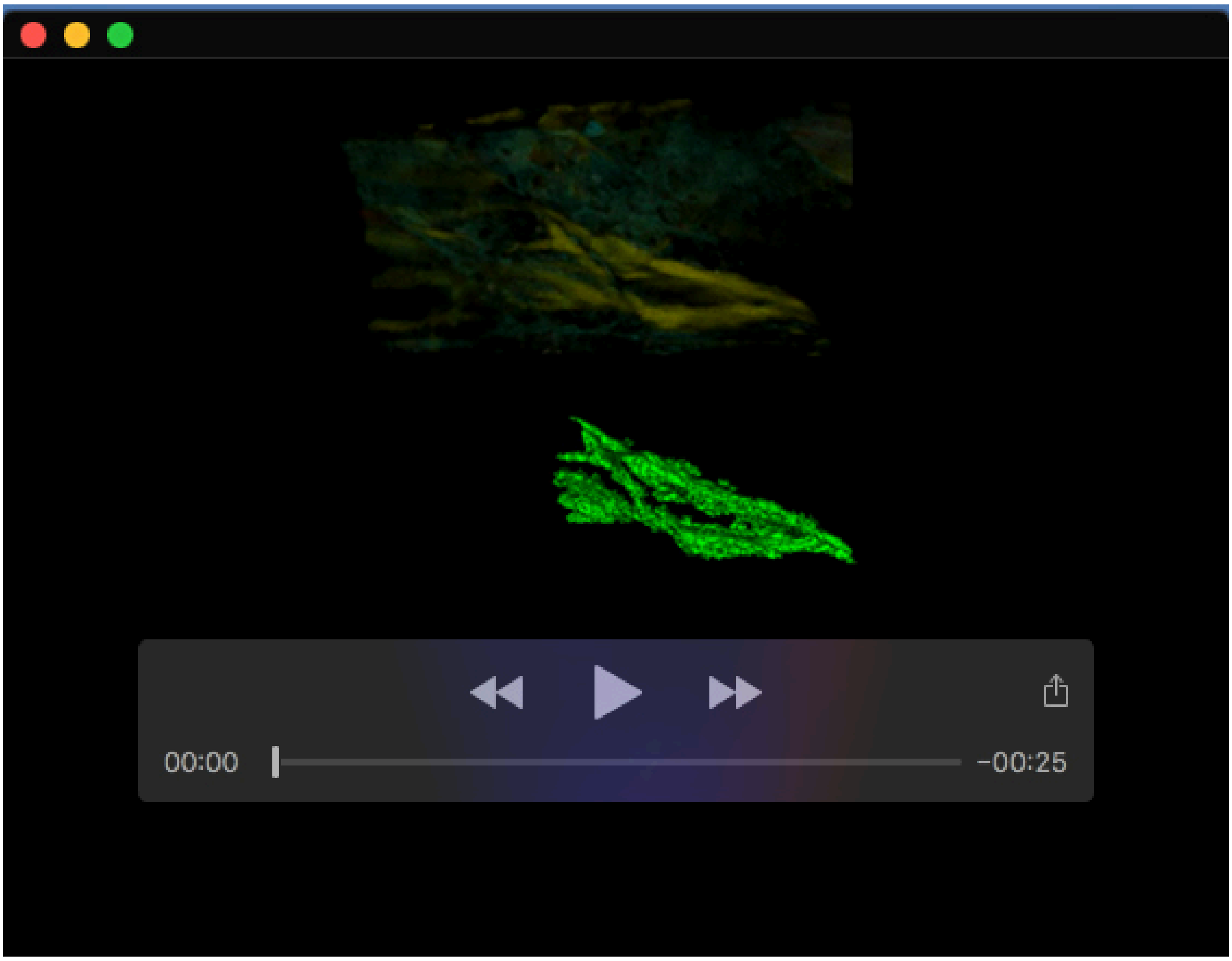

Movie 2. Rotation of healed wound.

Tg(BB3mus7)^{kg309};*Tg(HS:Cre.cryaa:RFP)*^{kg310} embryo was subjected to 5 min heat-shock at 24 hpf, a selected region containing a marked mnc wounded at 4 dpf and allowed to regenerate for 3 days while repeatedly observed (also shown in Fig. 6B-F). A 2-colour confocal stack at 3 dpw (top) was segmented (bottom) to reveal the marked-mnc-derived regenerated fibres in som14.
